# Supplementary material for: SyNDI: synchronous network data integration framework
Source: BMC Bioinformatics. 2018 Nov 6;19:403. doi: 10.1186/s12859-018-2426-5 (PMC6219086; doi:10.1186/s12859-018-2426-5)
Supplement: Supplementary file 5 — Files of the example presented in Additional file 4. This additional file is a zip package that contains all files used in Additional file 4. Detailed descriptions of each of these files are presented in a README file included in this zip package. (GZ 30411 kb) [file 12859_2018_2426_MOESM5_ESM.gz › SyNDITools/Meme2Fimo/meme_4.9.0_4.tar.gz!/meme_4.9.0/doc/iupac2meme.html]

iupac2meme


# iupac2meme

**Usage:** `iupac2meme [options] [<iupac_motif>]+`

**Description:**

Convert an IUPAC motif into a MEME version 4 formatted file
suitable for use with MAST and other MEME Suite programs.

**Input:**

An IUPAC motif represents frequencies by using either an exact letter
meaning that letter occurs in all sites, or ambiguous letters, representing
an equal frequency of all the letters representing by that letter. This
program additionally supports regular expression bracket expressions where
multiple letters can be grouped in one with the use of square brackets.

A background frequency file modifies the assumption of equal
probability of all alternative letters.

A probability matrix and optionally a log-odds matrix are output for each
motif provided on the command line. The probability matrix is computed using
pseudo-counts consisting of the background frequency (see -bg, below)
multiplied by the total pseudocounts (see -pseudo, below). The log-odds
matrix uses the background frequencies in the denominator and is log
base 2.

**Options:**

-alpha DNA|PROTEIN
:   IUPAC alphabet; default: DNA

-numseqs <numseqs>
:   assume frequencies based on this many sequences; default: 20

-bg <background file>
:   file with background frequencies of letters; default: uniform background

-pseudo <total pseudocounts>
:   add <total pseudocounts> times letter background to each frequency; default: 0

-logodds
:   output the log-odds (PSSM) and frequency (PSPM) motifs; default: PSPM motif only

-url <website>
:   website for the motif; a line with the tag
    'URL' followed by the provided website URL is added to the end of the output;
    default: no URL line

**Output:**

Writes MEME format to standard output.

**Sample Input:**

DNA IUPAC motif:

```
  ACGGWN[ACGT]YCGT
```

protein IUPAC motif:

```
  IKLVBZYXXHG
```
